# Significance of Nerve Plane for Inferior Mesenteric Plexus Preservation in Laparoscopic Rectal Cancer Surgery

**DOI:** 10.3389/fonc.2022.853662

**Published:** 2022-04-22

**Authors:** Kai Li, Junjie Zeng, Pengcheng Pang, Hua Cheng, Xiaobo He, Fengyu Cao, Qiang Luo, Shilun Tong, Yongbin Zheng

**Affiliations:** ^1^ Department of Gastrointestinal Surgery, Renmin Hospital of Wuhan University, Wuhan, China; ^2^ Division of Nephrology, Renmin Hospital of Wuhan University, Wuhan, China

**Keywords:** nerve plane, inferior mesenteric plexus, laparoscopic surgery, rectal cancer, station 253 node

## Abstract

**Background:**

Station 253 node dissection with high ligation of the inferior mesenteric artery (IMA) is difficult to perform without damage to the surrounding autonomic nerve plexuses. This study aimed to investigate the significance of the nerve plane for inferior mesenteric plexus (IMP) preservation in laparoscopic rectal cancer surgery.

**Methods:**

A total of 56 consecutive rectal patients underwent laparoscopic en bloc station 253 node dissection with high ligation of the IMA. Station 253 nodes were divided into the extra- and intra-nerve plane station 253 nodes for further H&E staining and immunohistochemical analysis. Based on IMP nerve plane-based evidence and histopathological results, a novel nerve-sparing technique, IMP nerve plane orientation, was proposed and performed on 68 rectal cancer patients. Urinary and sexual functions in all patients were evaluated at 6 months postoperatively.

**Results:**

Lymph node metastasis was not found, but abundant nerve bundles containing gangliocytes were observed in extra-nerve plane station 253 nodes. The nerve plane was identified intraoperatively and then confirmed by both postoperative gross specimen evaluation and histopathological analysis. The novel nerve-sparing technique (IMP nerve plane orientation) was successfully performed with no postoperative complications, and the operated patients had improved postoperative urinary and sexual functions.

**Conclusion:**

The nerve plane is helpful for IMP preservation and station 253 node dissection. This novel nerve-sparing technique of nerve plane orientation is technically feasible and safe, which could result in faster recovery of urinary and sexual functions.

## Introduction

The Japanese criteria practically define station 253 nodes as nodes that lie along the inferior mesenteric artery (IMA) from the origin of the left colic artery (LCA) to the origin of the IMA (1). The incidence of station 253 node metastasis is relatively low, i.e., approximately 0.3% to 8.6%, in different tumor stages (2). Station 253 node metastasis is more likely to occur in locally advanced rectal cancer above the peritoneal reflection (3); besides, en bloc station 253 node dissection is technically demanding and carries a risk of damage to the surrounding inferior mesenteric plexus (IMP), which may be associated with postoperative organ dysfunction, including urinary and sexual dysfunctions (4, 5). However, station 253 node metastasis is an important prognostic factor of rectal cancer. Therefore, whether en bloc station 253 node dissection is suitable for all rectal cancer patients remains controversial. Moreover, the current definition of station 253 nodes is limited to around the IMA, which is vague, and there is no consistent evidence about the clear border of the area of station 253 nodes.

The nerve plane was defined as the overlying tiny membranous tissue including the nerve, the adipose tissue, and extremely tiny capillaries around the nerve, as described in our previous studies (6–8). Similarly, there is still the IMP nerve plane around the IMA, which divides routine station 253 nodes into extra- and intra-nerve plane station 253 nodes. In the present study, we hypothesized that the nerve plane is the dorsal boundary of station 253 nodes, which should be preserved during station 253 node dissection; intra-nerve plane station 253 nodes are regional lymph nodes, which should be cleaned; and extra-nerve plane station 253 nodes are extra-mesenteric lymph nodes, which are unnecessarily cleaned. We believe that intraoperative IMP nerve plane preservation not only ensures that intra-mesenteric regional lymph nodes are cleaned totally but also better prevents damage to pelvic autonomic nerves. Therefore, the IMP nerve plane is clinically important in IMP preservation and station 253 node dissection in laparoscopic surgery for rectal cancer.

Therefore, the present study aimed to investigate the significance of the nerve plane for IMP preservation in laparoscopic rectal cancer surgery and to propose a novel nerve-sparing technique of nerve plane orientation for station 253 node dissection.

## Materials and Methods

**Figure 1:**
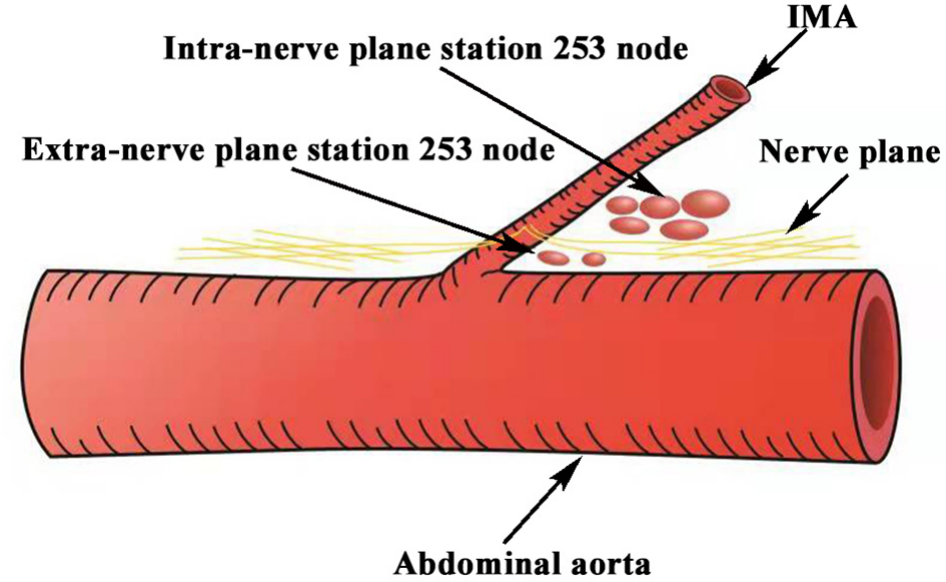
Schematic representation of the extra- and intra-nerve plane station 253 nodes. Station 253 nodes were divided into the extra- and intra-nerve plane station 253 nodes by the IMP nerve plane. IMA, inferior mesenteric artery; IMP, inferior mesenteric plexus.

A total of 124 rectal cancer patients were enrolled in this study; patients were divided into a nerve plane-oriented group and a no nerve plane-oriented group. From October 2019 to June 2020, a total of 56 rectal cancer patients in the no nerve plane-oriented group undergoing laparoscopic radical resection with en bloc station 253 node dissection in the Department of Gastrointestinal Surgery, Renmin Hospital of Wuhan University, were prospectively enrolled. Pelvic autonomic nerves including the IMP were preserved as much as possible, although en bloc station 253 nodes were cleaned. The IMP nerve plane was intraoperatively identified, and the routine station 253 nodes were divided into extra- and intra-nerve plane nodes (**Figure 1**). These lymph node tissues were dehydrated, paraffin embedded, cut into 5-µm sections, and processed for H&E staining. In addition, immunohistochemistry was performed to identify autonomic nerve fibers. Neuronal immunolabeling was performed with anti-rabbit Protein S100 antibodies (S100, 1:400; Abcam, Cambridge, MA, USA). S100 is a general immunolabeling marker of all nerves, which can specifically identify the nucleus and cytoplasm of Schwann cells in formalin-fixed paraffin-embedded nerve tissue specimens.

Based on IMP nerve plane-based evidence and histopathological examination, a novel nerve-sparing technique of nerve plane orientation for IMP preservation was performed on 68 rectal cancer patients in the nerve plane-oriented group between July 2020 and February 2021. Extra-nerve plane station 253 nodes were preserved, and intra-nerve plane station 253 nodes were cleaned and examined by H&E and immunohistochemical (S100) staining. All operations were performed by Prof. Yongbin Zheng, a chief physician of the Department of Gastrointestinal Surgery, Renmin Hospital of Wuhan University, with a 20-year post-certification experience performing approximately 350 cases of laparoscopic rectal cancer radical resection annually. Patient baseline data, tumor characteristics, and surgical outcomes were recorded, and all operative procedures were videotaped. Informed consent was obtained from all participating patients. This study was approved by the Institutional Review Board of Renmin Hospital, Wuhan University (no. WDRY2021-K126).

### Assessment of Sexual and Urinary Functions

The urinary function was evaluated by the International Prostate Symptom Score (IPSS) questionnaire (9). IPSS total score ranged from 0 to 35: the higher the score, the severer the urinary dysfunction. Moderate-to-severe urinary dysfunction was defined as an IPSS score >8 points (10). Male sexual function was evaluated by the 5-item version of the International Index of Erectile Function (IIEF-5) (11) questionnaire and ejaculation function grading. IIEF-5’s total score ranged from 1 to 25: the lower the score, the severer the erectile dysfunction. Male erectile dysfunction was defined as an IIEF-5 score ≤11 points. Ejaculation was functionally classified as follows: grade I, normal ejaculation; grade II, retrograde ejaculation; and grade III, anejaculation. Ejaculation dysfunction was identified as grade II or III ejaculation in this study (12). Female sexual function was evaluated by the Female Sexual Function Index (FSFI) system, which is a validated questionnaire comprised of 19 items. FSFI’s total score ranged from 2 to 36; the higher the score, the severer the sexual dysfunction (13). Female sexual dysfunction was defined as an FSFI score <26.55 points in this study (14). Urinary and sexual functions were evaluated before surgery and at 6 postoperative months.

### Statistical Analysis

Data were presented as mean ( ± SD) and number (frequency, %) for continuous and categorical variables, respectively. Dichotomous clinical variables were assessed by a chi-square test. Two-sided *p* < 0.05 was considered statistically significant. All statistical analyses were performed with SPSS version 20.0 (IBM, Armonk, New York, NY, USA).

## Results

### Clinical Characteristics

A total of 124 rectal cancer patients were evaluated in this study. All participating patients received laparoscopic radical resection without conversion. Patient clinical characteristics are shown in **Table 1**. Variables, including age, sex, body mass index (BMI), prostatic hyperplasia or not, tumor location, tumor differentiation, pathological TNM stage, intra-nerve plane station 253 nodes retrieved, and total lymph nodes retrieved, had no significant differences between the two groups. Patients in the nerve plane-oriented group had reduced operative blood loss (49.6 ± 13.6 vs. 22.6 ± 13.7, *p* < 0.001) but longer operative time (165.2 ± 20.6 vs. 175 ± 25.7, *p* = 0.016) compared with patients in the no nerve plane-oriented group.

**Table 1:** Clinical and pathological characteristics of participating patients.

Variable	No nerve plane-oriented group (N = 56)	Nerve plane-oriented group (N = 68)	*p*
Age, mean ± SD	58.9 ± 8.4	56.0 ± 8.6	0.067
Sex (male/female)	31/25	35/33	0.666
Body mass index, kg/m^2^, mean ± SD	24.3 ± 2.4	25.2 ± 3.1	0.054
History of prostatic hyperplasia, n (%)	3 (5.4%)	2 (2.9%)	0.496
Tumor site, n (%)			0.587
Above peritoneal reflexes	34 (60.7%)	38 (55.9%)	
Below peritoneal reflexes	22 (39.3%)	30 (44.1%)	
Tumor differentiation, n (%)			0.425
Well or moderate	40 (71.4%)	44 (64.7%)	
Poor, mucinous or signet-ring cell	16 (28.6%)	24 (35.3%)	
Pathological TNM stage^*^, n (%)			0.835
I	14 (25.0%)	14 (20.6%)	
II	29 (51.8%)	38 (55.9%)	
III	13 (23.2%)	16 (23.5%)	
Estimated blood loss, ml, mean ± SD	49.6 ± 13.6	22.6 ± 13.7	0.000
Total operative time, min, mean ± SD	165.2 ± 20.6	175 ± 25.7	0.016
Intra-nerve plane station 253 node retrieved, mean	3.0 ± 1.4	3.1 ± 1.2	0.707
Positive intra-nerve plane station 253 nodes, n (%)	5 (8.9%)	6 (8.8%)	0.984
Extra-nerve plane station 253 nodes retrieved, mean	1.5 ± 0.8		
Positive extra-nerve plane station 253 nodes, n (%)	0		
Total lymph nodes retrieved, mean ± SD	20.7 ± 6.4	22.2 ± 8.9	0.299
Total positive lymph nodes, n (%)	13 (23.2%)	16 (23.5%)	0.967

SD, standard deviation.

^*^TNM stage according to the American Joint Committee on Cancer, 7th ed.

### Nerve Plane and Lymph Nodes

**Figure 2:**
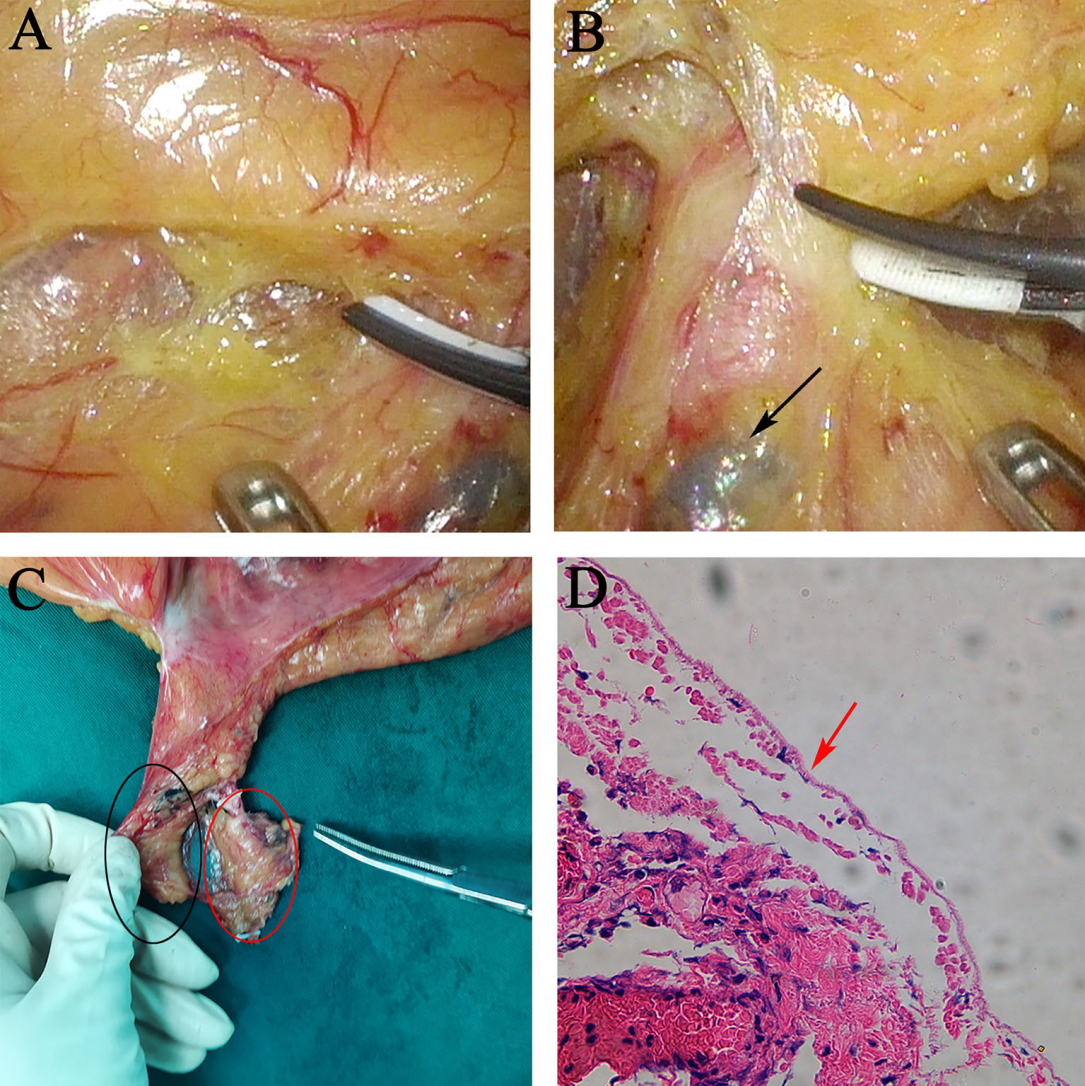
Relationship between extra- and intra-nerve plane station 253 nodes. **(A)** Inferior mesenteric plexus nerve plane. The IMP was surrounded by the adipose tissue and extremely tiny capillaries and covered by a tiny membranous tissue. **(B)** Extra-nerve plane station 253 nodes were black-dyed by carbon nanoparticles. **(C)** Postoperative gross specimen of en bloc station 253 nodes. Black and red ovals show intra- and extra-nerve plane station 253 nodes, respectively. **(D)** H&E staining showing completely excised mesorectum of intra-nerve plane station 253 nodes; no nerve bundles were found in intra-nerve plane station 253 nodes. IMA, inferior mesenteric artery; IMP, inferior mesenteric plexus.

In all patients, the typical structural features of the nerve plane were observed, as shown in **Supplementary Video 1**. The “Holy plane” was revealed more clearly in the anterior of the abdominal aorta by enforcing traction and anti-traction, and the IMP was lifted by the IMA as a “tent” when the extension was closed to the root of the IMA; the IMP was surrounded by the adipose tissue and extremely tiny capillaries and covered by a tiny membranous tissue, i.e., the IMP nerve plane (**Figure 2A**). Similarly, the abdominal aortic plexus and superior hypogastric plexus nerve planes were also observed. These nerve planes are continuous and form a novel landmark for pelvic autonomic nerve protection in laparoscopic rectal cancer surgery. Station 253 nodes within the nerve plane are called intra-nerve plane station 253 nodes, which should be cleaned in rectal cancer surgery; conversely, station 253 nodes beyond the nerve plane are called extra-nerve plane lymph nodes, which are rarely involved in tumor metastasis due to the protective effect of the nerve plane (**Figure 2B**). Subsequently, en bloc station 253 nodes were marked and divided into extra- and intra-nerve plane nodes after the specimen was removed (**Figure 2C**), which were sent to a pathological lab for further examination. Eventually, the mean number of intra-nerve plane station 253 nodes retrieved was 3.0 (range, 0–7), and 5 cases (5/56 = 8.9%) with intra-nerve plane station 253 node metastasis were confirmed by H&E staining. The mean number of extra-nerve plane station 253 nodes retrieved was 1.5 (range, 0–4), and no metastatic extra-nerve plane station 253 nodes were found. In the nerve plane-oriented group, the mean number of intra-nerve plane station 253 nodes retrieved was 3.1 (range, 1–6), and 6 cases (6/68 = 8.8%) showed intra-nerve plane station 253 node metastasis, as shown in **Table 1**.

### Histopathological Results

**Figure 3:**
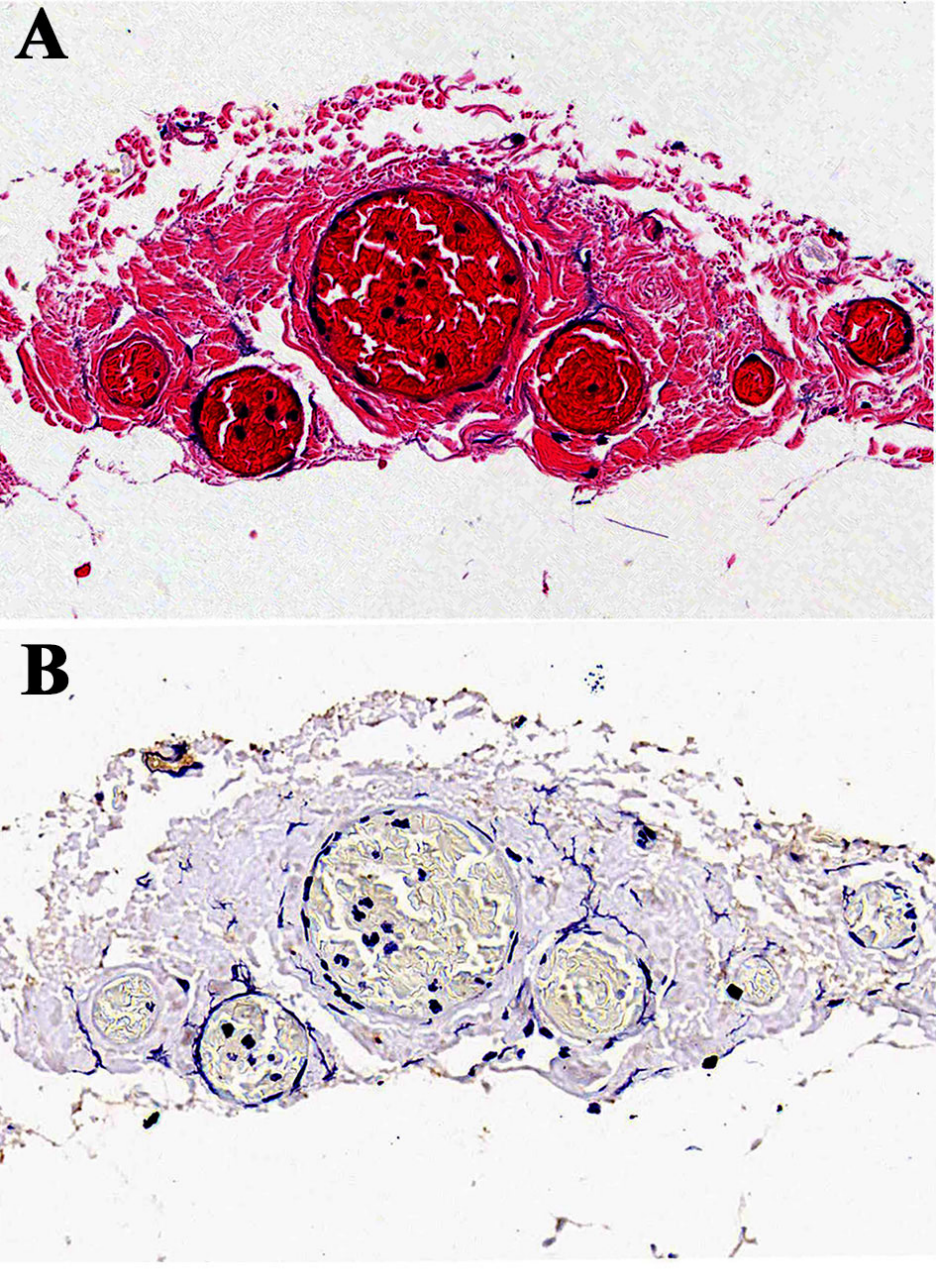
H&E and immunohistochemical staining (S100) of extra-nerve plane station 253 nodes. **(A)** H&E staining of extra-nerve plane station 253 nodes. **(B)** Immunohistochemical staining of extra-nerve plane station 253 nodes with anti-S100 antibodies. Abundant nerve fibers positively stained for S100 were deep brown and observed in extra-nerve plane station 253 nodes.

Nerve fibers positively stained for S100 were deep brown, and nerve bundles containing gangliocytes were observed in extra-nerve plane station 253 nodes (**Figure 3**). No nerve bundles were found in intra-nerve plane station 253 nodes, and the mesorectum in postoperative specimens was intact as shown by H&E staining (**Figure 2D**).

### Nerve Plane-Based Surgical Technique

**Figure 4:**
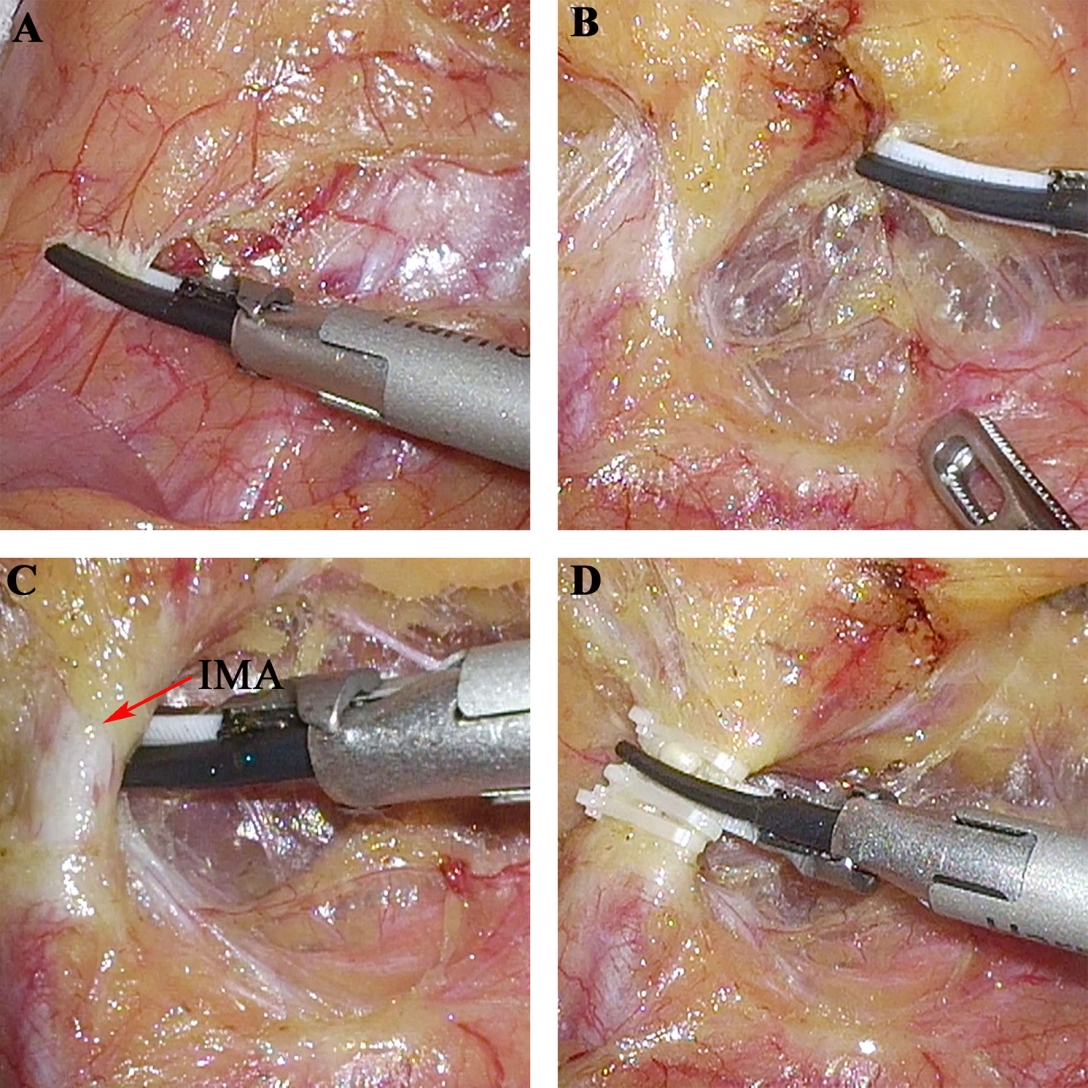
The nerve-sparing technique of nerve plane-oriented. **(A)** The dissection extended up to the lower edge of the duodenum. **(B)** Extension from the superior hypogastric plexus nerve plane to the IMP nerve plane posterior to the IMA. **(C)** Abdominal aortic plexus nerve plane was gradually entered from cephalic to caudal by the “slope downhill” approach. **(D)** The IMA was ligated and cut above the IMP nerve plane. IMA, inferior mesenteric artery; IMP, inferior mesenteric plexus.

Based on the above nerve plane-based evidence and histopathological results, a novel nerve-sparing technique of nerve plane orientation was proposed. The details of the surgical procedure are shown in **Supplementary Video 2**. Briefly, dissection above the abdominal aortic plexus nerve plane was performed and extended up to the lower edge of the duodenum and down to the entrance of the IMP nerve plane (**Figure 4A**). After extension from the superior hypogastric plexus nerve plane to the IMP nerve plane posterior to the IMA (**Figure 4B**), dissection was continued over the IMP nerve plane from caudal to cephalic by the “slope climbing” approach. After crossing the IMA, dissection was continued from cephalic to caudal by the “slope downhill” approach, and the abdominal aortic plexus nerve plane was gradually entered (**Figure 4C**). Finally, the IMA was ligated and cut above the IMP nerve plane (**Figure 4D**). After a specimen was collected, the nerve plane around the IMA showed preserved integrity.

### Urinary and Sexual Functions

As shown in **Table 2**, there were no significant differences in IPSS (4.3 ± 2.9 vs. 5.2 ± 2.5, *p* = 0.062), IIEF-5 (16.5 ± 3.4 vs. 17.7 ± 3.7, *p* = 0.132), and FSFI (29.2 ± 3.8 vs. 29.5 ± 3.7, *p* = 0.638) scores before surgery between the two groups. Patients in the nerve plane-oriented group showed higher IIEF-5 (13.9 ± 3.1 vs. 15.8 ± 3.7, *p* = 0.015) and FSFI (24.6 ± 4.6 vs. 27.7 ± 3.1, *p* = 0.006) scores than those in the no nerve plane-oriented group, although IPSS scores (6.4 ± 4.7 vs. 6.0 ± 3.4, *p* = 0.635) showed no significant difference between the two groups at 6 postoperative months.

**Table 2:** Urinary and sexual function of participating patients.

**Variable**	**No nerve plane-oriented group** **(N = 56)**	**Nerve plane-oriented group** **(N = 68)**	***p***
**IPSS**, mean ± SD			
Preoperatively	4.3 ± 2.9	5.2 ± 2.5	0.062
6 months postoperatively	6.4 ± 4.7	6.0 ± 3.4	0.635
**IIEF-5**, mean ± SD			
Preoperatively	16.5 ± 3.4	17.7 ± 3.7	0.132
6 months postoperatively	13.9 ± 3.1	15.8 ± 3.7	0.015
**FSFI**, mean ± SD			
Preoperatively	29.2 ± 3.8	29.5 ± 3.7	0.638
6 months postoperatively	24.6 ± 4.6	27.7 ± 3.1	0.006
**Urinary dysfunction**, n (%)			
Preoperatively	5 (8.9%)	5 (7.3%)	0.748
6 months postoperatively	15 (26.8%)	8 (11.7%)	0.032
**Sexual dysfunction**, n (%)			
Preoperatively	7 (12.5%)	9 (13.2%)	0.903
6 months postoperatively	20 (35.7%)	13 (19.1%)	0.037

SD, standard deviation.

IPSS, International Prostate Symptom Score; IIEF-5, 5-item version of the International Index of Erectile Function; FSFI, Female Sexual Function Index.

There were no significant differences in urinary (8.9% vs. 7.3%, *p* = 0.748) and sexual (12.5% vs. 13.2%, *p* = 0.903) dysfunction rates before surgery between the two groups. However, patients in the nerve plane-oriented group had lower urinary (26.8% vs. 11.7%, *p* = 0.032) and sexual (35.7% vs. 19.1%, *p* = 0.037) dysfunction rates than those in the no nerve plane-oriented group at 6 postoperative months. Therefore, the patients who underwent the nerve-sparing technique of nerve plane orientation had better postoperative urinary and sexual functions.

## Discussion

With the introduction of total mesorectal excision (TME) surgery, rectal cancer prognosis has been greatly improved; however, the quality of life of patients after surgery has not been paid enough attention to by surgeons, which includes urinary, sexual, and bowel dysfunctions related to intraoperative pelvic autonomic nerve injury. Therefore, preserving the pelvic autonomic nerve, including IMP preservation, in rectal cancer surgery has become increasingly important. Many studies have found that station 253 node metastasis is an important prognostic factor in patients with colorectal cancer (15–17). Many consensus opinions, including the Japanese Society for Cancer of the Colon and Rectum (JSCCR) and The American Society of Colon and Rectal Surgeons Clinical Practice Guidelines, recommended station 253 node dissection and high ligation of the IMA in rectal cancer patients with preoperative clinical T_2–4_N_+_ stage (18, 19). However, high ligation of the IMA and station 253 node dissection in laparoscopic anterior resection for rectal cancer increase the risk of IMP damage and result in postoperative urinary and sexual dysfunctions. Therefore, how to improve surgical techniques for IMP preservation is essential and represents a technical challenge for most surgeons; surgical techniques for IMP preservation are still lacking.

In the present study, the nerve plane was used as an optimal surgical landmark for laparoscopic rectal surgery, for several potential reasons. First, pelvic autonomic nerves, including the IMP, are located outside the mesorectal plane (20, 21); therefore, this technique should theoretically have consistent oncologic outcomes with routine nerve-sparing surgery. Second, not only the nerves but also the continuous nerve plane covered with the membranous tissue are preserved; thus, it could reduce intraoperative damage to pelvic autonomic nerves due to heat conduction and physicochemical factors such as wound exudate and inflammatory mediators. Finally, the nerves were not intentionally exposed during the operation, which differs from other nerve-sparing techniques such as nerve-guided technique (22, 23), active exposure of the nerve could increase nerve stretching and the risk of nerve damage. The nerve-sparing technique of nerve plane orientation for IMP preservation has been a standardized laparoscopic procedure in our institution since 2013, with satisfactory postoperative urinary and sexual functions despite the lack of follow-up for survival prognosis.

Currently, numerous studies have proposed various nerve-sparing techniques for IMP preservation. Liang et al. (24) recommended that the IMP should be preserved by sparing the pre-aortic connective tissue and leaving a 1- to 2-cm-long stump of the IMA *in situ*, consistent with most nerve-sparing techniques (25, 26). Huscher et al. (27) reported a clear identification of the branches climbing around the IMA with the Cavitron ultrasonic surgical aspirator for IMP preservation; while performing a high ligation, this nerve-sparing technique relies more on advanced medical devices than surgical technology. Sun et al. (28) also reported three anatomical levels, including the parietal fascia, the neurofascial layer, and the mesosigmoid, for IMP preservation in laparoscopic rectal cancer surgery and proposed an effective procedure for high ligation of the IMA. The latter technique was somewhat similar to ours; however, the neurofascial layer could not form a continuous plane in their surgical procedure, as shown in the current technique. Recently, Zheng et al. (29) reported that the left trunk of the IMP is a part of the IMA vascular sheath and proposed intrasheath separation of the IMA and left trunk for IMP preservation. In the present study, we believed that the IMP nerve plane could be separated from the IMA by enforcing traction and anti-traction. To maintain a balance between oncology and function, the nerve-sparing technique of nerve plane orientation was successfully performed in 68 patients without postoperative complications or urinary/sexual dysfunction. Therefore, this surgical technique is technically safe and feasible.

Moreover, the clear border of station 253 nodes remains vague and not standardized, and the area of station 253 nodes is regarded as an area containing the fat tissue around the IMA. Studies recommended that the left side is the inferior mesenteric vein (IMV), with the side dissection being the abdominal aorta, the cephalic side being the duodenum, and the caudal side being the IMA, which are the borders of station 253 nodes (30). In addition, most Chinese experts believe that the borders of station 253 nodes include the medial border as the area of the trunk between the point of origin of the IMA and the LCA, the lateral border as the medial margin of the IMV, the caudal border as the point of origin of the LCA to its intersection with the IMV, and the cephalic border as the level of the root of the IMA (31). However, most studies have no clear definition of the dorsal border of station 253 nodes. In the present work, we believed that the IMP nerve plane is the dorsal border of station 253 nodes and should be preserved, which not only avoids damaging the IMP but also ensures station 253 node dissection in laparoscopic rectal cancer surgery.

Regarding urinary and sexual functions, in the present study, at 6 months after laparoscopic rectal cancer surgery, with the routine nerve-sparing technique, the rate of urinary dysfunction was increased by 17.9% (from 8.9% to 26.8%), while the sexual dysfunction rate was increased by 23.2% (from 12.5% to 35.7%). However, in the nerve-sparing technique of nerve plane orientation, the rate of urinary dysfunction was increased by 4.4% (from 7.3% to 11.7%), and the sexual dysfunction rate was increased by 5.9% (from 13.2% to 19.1%). Therefore, the nerve-sparing technique of nerve plane orientation confers faster recovery of urinary and urogenital functions.

In the present study, we regarded extra-nerve plane station 253 nodes as extra-mesenteric lymph nodes, which are unnecessarily cleaned, especially for patients with early rectal cancer. Extended station 253 node dissection not only provides no additional survival benefit but also increases the risk of nerve damage. However, some limitations should be acknowledged in this study, including the relatively small sample size and the lack of long-term follow-up for oncological outcomes. Therefore, further studies with large samples and long-term follow-up with oncological outcomes are required.

## Conclusion

The presence of the nerve plane is helpful for IMP preservation. This nerve-sparing technique of nerve plane orientation is technically feasible and safe, which confers faster recovery of urinary and sexual functions.

## Data Availability Statement

The raw data supporting the conclusions of this article will be made available by the authors, without undue reservation.

## Ethics Statement

The studies involving human participants were reviewed and approved by The Institutional Review Board of the Renmin Hospital of Wuhan University. The patients/participants provided their written informed consent to participate in this study.

## Author Contributions

KL designed the study and prepared the manuscript draft. PP, HC, and JZ performed the data collection and analysis. QL and ST performed the H&E staining and immunohistochemical analysis. XH, FC, and YZ critically revised the manuscript for important intellectual content. All authors contributed to the article and approved the submitted version.

## Funding

This work was supported by grants from the Chenxiao-ping Foundation for the Development of Science and Technology of Hubei Province (CXPJJH121003-2103).

## Conflict of Interest

The authors declare that the research was conducted in the absence of any commercial or financial relationships that could be construed as a potential conflict of interest.

## Publisher’s Note

All claims expressed in this article are solely those of the authors and do not necessarily represent those of their affiliated organizations, or those of the publisher, the editors and the reviewers. Any product that may be evaluated in this article, or claim that may be made by its manufacturer, is not guaranteed or endorsed by the publisher.
